# Biomechanical assessment of the ipsilesional upper limb in post-stroke patients during multi-joint reaching tasks: A quantitative study

**DOI:** 10.3389/fresc.2022.943397

**Published:** 2022-07-28

**Authors:** Alessandro Scano, Eleonora Guanziroli, Robert M. Mira, Cristina Brambilla, Lorenzo Molinari Tosatti, Franco Molteni

**Affiliations:** ^1^Institute of Intelligent Industrial Technologies and Systems for Advanced Manufacturing (STIIMA), Italian National Research Council (CNR), Milan-Lecco, Italy; ^2^Villa Beretta Rehabilitation Center, Costa Masnaga, Italy

**Keywords:** stroke, upper-extremity, biomechanics, ipsilesional, dynamics, motor control

## Abstract

In hemiplegic patients with stroke, investigating the ipsilesional limb may shed light on the upper limb motor control, impairments and mechanisms of functional recovery. Usually investigation of motor impairment and rehabilitative interventions in patients are performed only based on the contralesional limb. Previous studies found that also the ipsilesional limb presents motor deficits, mostly evaluated with clinical scales which could lack of sensibility. To quantitatively evaluate the performance of the ipsilesional limb in patient with stroke, we conducted an observational study in which 49 hemiplegic patients were enrolled, divided in subgroups based on the severity of impairment of the contralesional limb, and assessed with a kinematic, dynamic and motor control evaluation protocol on their ipsilesional upper limb during reaching movements. Measurements were repeated in the acute and subacute phases and compared to healthy controls. Our results showed that the ipsilesional limb presented lower kinematic and dynamic performances with respect to the healthy controls. Patients performed the movements slower and with a reduced range of motion, indicating a difficulty in controlling the motion of the arm. The energy and the power outputs were lower in both shoulder and elbow joint with a high significance level, confirming the limitation found in kinematics. Moreover, we showed that motor deficits were higher in the acute phase with respect to the subacute one and we found higher significant differences in the group with a more severe contralesional limb impairment. Ipsilesional upper limb biomechanics adds significant and more sensible measures for assessments based on multi-joints dynamics, providing a better insight on the upper limb motor control after stroke. These results could have clinical implications while evaluating and treating ipsilesional and contralesional upper limb impairments and dysfunctions in patients with stroke.

## Introduction

Stroke is one of the major leading cause of death worldwide and it is the third cause of disability in adults: it is estimated that stroke affects about 795,000 people every year (1). For post-stroke survivors, the physical, psychological and financial consequences strongly affect patients, their families and society (2). The functional limitations in patients with stroke are mainly related to deficits in the contralesional side of the body such as impaired control of multi-joint movements coordination, muscular weakness or spasticity (3). Furthermore, unilateral stroke limits the motor function of the ipsilesional side, which is generally considered as “unaffected” (4). Although ipsilesional deficits are less severe than contralesional, they could impact negatively on the performance of daily activities of stroke survivors. Understanding the role of the damaged brain hemisphere on the ipsilesional side motor control may support the global functional recovery of patients (5). Nevertheless, potential causes of the role of a hemisphere to the ipsilesional movement have been hypothesized. First, while most of the anatomical pathways of corticospinal motor fibers cross to the opposite side in the spinal cord, 10–15% of fibers descend in the same side of the cortical origin (6). It follows that each hemisphere plays a role in controlling the movements of both sides. Moreover, the interconnection between the two hemispheres *via* the corpus callosum provokes that the contralateral hemisphere is involved to modulate the activation of the ipsilateral one (7). Lastly, the contralateral motor activity acts as an efferent copy of the ipsilateral limb state (5); if the contralateral efference copy is missing or reduced, the ipsilateral limb can be affected as well.

Previous studies have confirmed that the ipsilesional side shows deficits in dexterous tasks (8), in movement coordination (9) and in wrist movements (10) when comparing the motor performance to healthy subjects. Ipsilesional impairment is mainly related to movement speed and smoothness (11) and is maximum immediately after the stroke event, even if motor performance improves in time (8, 11). In fact, patients have shown slower movements in the ipsilesional upper extremities (12), probably due to an increased cognitive demand in sequencing the movement (13). Moreover, greater ipsilesional deficits are present in patients with a major extension of the lesion in the contralateral hemisphere (14, 15). These studies demonstrated the impairment of the ipsilesional limb and most of them use clinical scales for the evaluation, offering a great variety of assessments. However, these scales could have some biases due to the operator and they are sensible only for gross motor improvements (16). A biomechanical assessment provides a quantitative evaluation of the performance, including more objectivity and higher sensibility, as already shown in kinematic analysis of the contralesional limb (17, 18). Quantifying the biomechanics of the ipsilesional upper limb function post-stroke paves the way for implementing specific rehabilitation programs, extending traditional approaches a focus on both the contralesional and ipsilesional upper limbs (19, 20).

The aim of this study was to provide a quantitative assessment of the ipsilesional upper limb of patients with stroke performing frontal reaching movement. Patients were evaluated in acute and subacute stage, comparing kinematic, dynamic and motor control variables to healthy controls.

## Materials and methods

### Study design

We conducted an observational study in which kinematic, dynamic and motor control data of the ipsilesional limb of stroke patients were compared to age-matched controls performing reaching movement, a multi-joint movement requiring refined coordination (21). Patients were assessed at two different post-stroke phases (acute and subacute stage) in order to evaluate the motor impairment at different times after the stroke event. To investigate the existence of a correlation between the severity of motor performance of the contralesional and the ipsilesional, patients were divided in groups based on their motor capacity.

### Participants

Two cohorts of participants were included in this study. Eligible post ischemic patients with stroke, with unilateral upper limb deficit from Villa Beretta Rehabilitation Center, Ospedale Valduce, Costa Masnaga, Italy were recruited. Inclusion criteria were: ischemic stroke survivors; unilateral upper limb deficit; ability to understand the instructions and ability to remain in a sitting posture. Exclusion criteria were: bilateral impairment; cognitive impairment; other severe medical problems. Patients were evaluated at T0—acute stage (<15 days from the acute event) and at T1—subacute stage (about 45 days after T0) and were divided into three subgroups based on the upper limb evaluation with the total Motricity Index (22) administered by an experienced physician: severe (MI ≤ 30); moderate (30 < MI ≤ 70) and mild (MI > 70). Healthy control participants were included if they were age-matched and without neurological or musculoskeletal impairments. Prior to testing, all healthy subjects were questioned and clinically evaluated for the presence of neurological or orthopedic signs, and excluded if any.

Ethical approval was granted by the local ethical committee at ATS Insubria and the experimental trial was conducted in compliance with the Declaration of Helsinki (23). All healthy people and patients have given their informed consent for participation in the research study. Both healthy subjects and patients were then instrumented with markers (24, 25) by an experienced bioengineer.

Since patients were expected to have lower performances with respect to the control group, relatively low subjects are needed due to the large effect size expected. To determine the number of subjects to be enrolled, we used the software GPower 3.1.9.7 software (Heinrich Heine University, Dusseldorf, Germany). A clinically relevant threshold was imposed for each measure in order to catch a functional difference between patients and controls (e.g., 8° for shoulder elevation required 11 samples per group) and the GPower a priori test was repeated for all the measures included in the work always leading to a minimum range from 8 to 15 subjects per group.

### Data collection procedure

**Figure 1:**
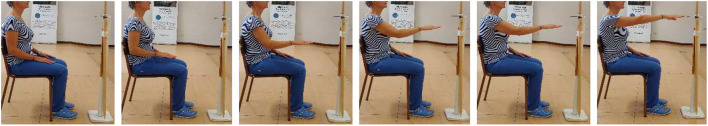
Lateral view of arm reaching performed by a healthy subject.

Healthy people and patients followed a protocol presented in recent studies (24, 25). The subjects sat on a chair, adjustable for height, with the feet resting on the floor and the knees and hips bent at 90 degrees. In the rest position, both hands were lying on the thighs, and the arms were positioned with flexed elbow and slightly extended shoulder. Starting from rest position, subjects were asked to carry out the movements without moving his/her back away from the backrest. To perform the reaching movements, each subject had to move the hand toward a target located in front of the subject at shoulder height, at a distance slightly longer than that of the fully extended upper limb, as shown in Figure 1. Each subject performed 20 repetitions of the reaching movement.

Tracking data were collected with a 3-dimensional (3D) optoelectronic motion tracking system (8 TVc 250 Hz; SMART BTS, Italy). In order to limit the overall setup time and facing the stringent requirements of the clinical practices, and considering further exploitations with low cost devices such as Kinect (18), 5 markers were used to track the arm kinematics. Markers were applied to the spinous process of D5 (M1), the spinous process of C7 (M2), acromion (M3), lateral epicondyle of the elbow (M4), and styloid process of the ulna (M5). The target marker M6 was placed in front of the subject slightly exceeding reaching distance.

In this study, measurements for patients were repeated at T0—acute stage (less than 15 days after the acute event) and subacute stage T1 (about 45 days after T0). We assessed whether the ipsilesional limb was comparable to healthy, both at T0 and T1. For controls, measurements were acquired in one session. The study took place at Villa Beretta Rehabilitation Center, Ospedale Valduce, Costa Masnaga, Italy.

### Data analysis procedure

The evaluation protocol extended previous works (24), including a 3D dynamic model (26). All data analysis and pre-elaborations were performed offline in MATLAB (Mathworks, Natick, MA, USA).

In the pre-processing stage, all marker coordinates were filtered with a 3rd order low-pass Butterworth filter with a cut-off frequency of 6 Hz, in order to remove noise and movement artifacts (27). Each trial was segmented in subtasks, each one beginning when the subjects started to elevate the arm until they reached the target. The starting and the ending points were identified with a threshold algorithm applied to the velocity profiles of the shoulder flexion in the sagittal plane. All the parameters of the assessment were computed for each task separately and then collected for the statistical analysis. Our outcome measures included several biomechanical parameters.

The task execution time *TE* was computed, as the time required to perform each task, between each movement starting and ending. The time to peak velocity *TP* (28) was calculated as:


(1) $$\begin{array}{l} TP=\frac{t_{0}-t_{vp}}{TE} \end{array}$$

Where *t*_0_ is the task initiation time and *t*_*vp*_ the time in which the velocity peak occurs.

Movement smoothness was evaluated with the normalized jerk *NJ* (29), computed as follows:


(2) $$\begin{array}{l} NJ=\sqrt{\frac{1}{2}\cdot \frac{t_{tot}^{5}}{L^{2}}\cdot \int j^{2}\;dt} \end{array}$$

Where *t*_*tot*_ is the task execution time, *j* is the third derivative of the wrist 3D trajectory and *L* is the length of the wrist trajectory during the execution of the task. The upper limb configuration was then assessed using two clinical angles: shoulder flexion (_SF_) and elbow flexion (_EF_). For both _SF_ and _EF_ we evaluated:

- The maximum angular displacement (*MAX*_*SF*_, *MAX*_*EF*_), as the maximum angle reached at the end of the task;
- The minimum angular displacement (*MIN*_*SF*_, *MIN*_*EF*_), as the minimum angle at the beginning of the task;
- The range of motion (*ROM*_*SF*_, *ROM*_*EF*_), as the difference between the maximum and the minimum angle.

Using the weight and height of each subject and anthropometric tables (30), the inverse dynamics of the upper limb was computed through Euler-Newton equations to compute joint torques. Moreover, the analysis was extended to the computation of the exerted power *P*_*i*_ and expended energy *E*_*i*_ for each joint *i*; moreover, to permit inter subject analysis, all dynamic parameters were normalized to the length of the subject's arm (*La*) and weight (*Ma*).


(3) $$\begin{array}{l} {\bar{\tau }}_{i}\;(t)=\frac{\tau_{i}\;(t)}{La\cdot Ma} \end{array}$$

(4) $$\begin{array}{l} P_{i}\;(t)={\bar{\tau }}_{i}\;(t)\cdot \omega_{i}\;(t) \end{array}$$

Where τ_*i*_ is the torque, τ_*i*_ is the normalized torque and ω_*i*_ is the angular velocity of the joint *i*. In order to evaluate the maximum effort exerted by each subject, we considered the peak power *P*_*MAX*_, computed as the maximum value of the power *P* time series for shoulder (*P*_*SF*_) and elbow (*P*_*EF*_).

Finally, the normalized expended energy *E*_*SF*_ and *E*_*EF*_ for shoulder flexion and elbow flexion were computed as follows:


(5) $$\begin{array}{l} E_{i}=\int_{t_{0}}^{t_{end}}P_{i}\;(t)\;dt \end{array}$$

Where *t*_0_ and *t*_*end*_ are the initial and the ending time of the task, and *P*_*i*_*(t)* is the power time course calculated in equation (4). Only the normalized power and energy were considered in the comparison.

### Statistical analysis

All data distributions were tested for normality through the Kolmogorov-Smirnov test. As the results for all parameters of interest were normally distributed, a parametric statistic test was used. For all the proposed variables of the assessment (kinematic and dynamic), a two-sample *t*-test was computed to compare each subgroup of patient (severe, moderate, mild) at each stage (T0 and T1) to the control group, performing a total of six tests for each variable. The significance level was set at *p* < 0.05. Statistical analyses were performed using Matlab2021b software (MathWorks, Natick, MA, USA).

## Results

**Figure 2:**
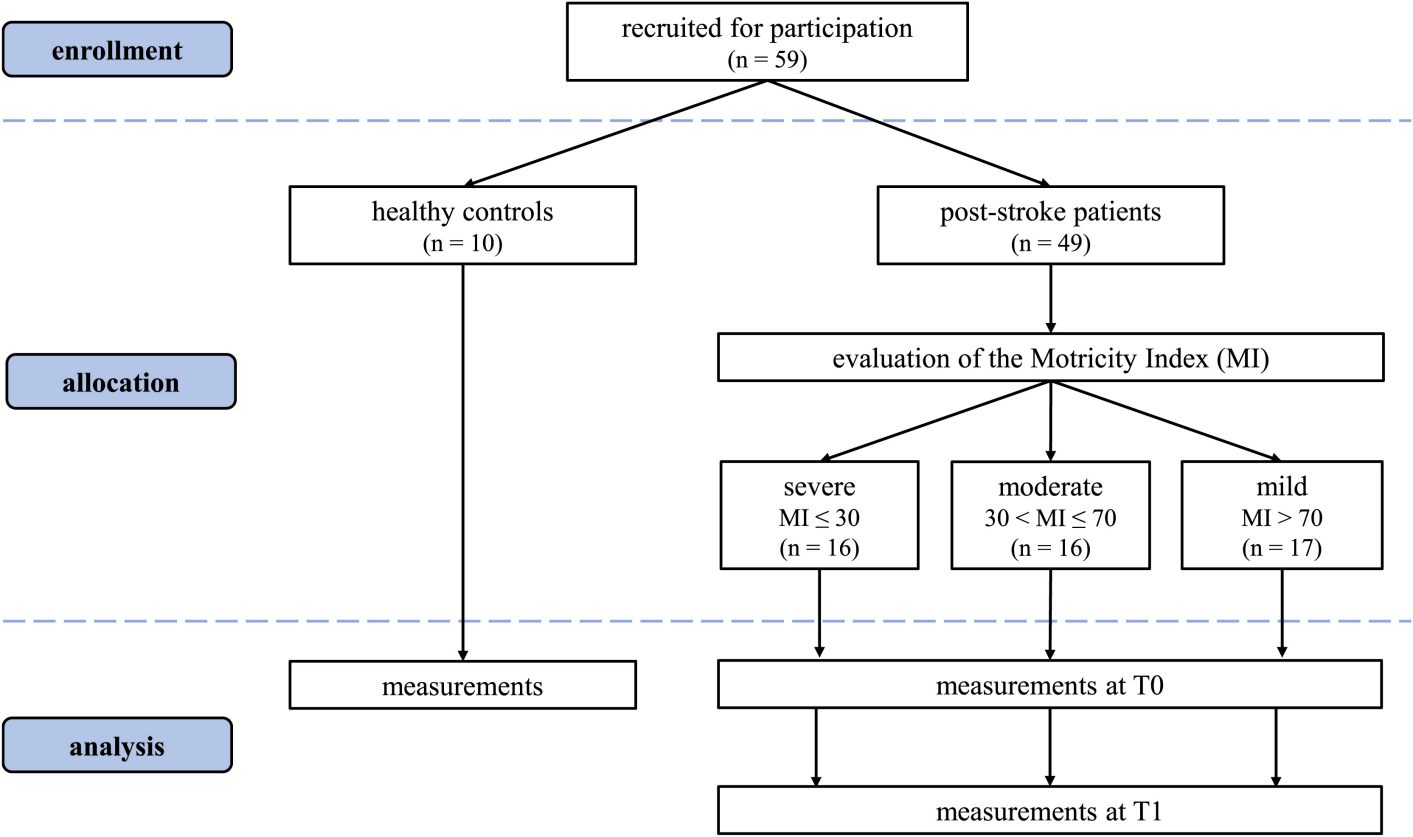
Consolidated Standards of Reporting Trials (CONSORT) flow diagram.

A total of 49 patients was enrolled in this study: 16 in the severe subgroup [10F, 6M, 71.9 (8.1) years, 68.4 (8.3) kg, 166.5 (7.7) cm]; 16 in the moderate [2F, 14M, 66.3 (12.5) years, 75.3 (11.2) kg, 170.4 (8.3) cm] and 17 in the mild one [8F, 9M, 65.9 (11.9) years, 71.53 (8.9) kg, 169.8 (9.9) cm]. From healthy age-matched control subjects, 20 limbs were included [5F, 5M, age 64 (8) years, weight 62 (10) kg, height 170.0 (10) cm]. The CONSORT flow diagram (31) for the study is shown in Figure 2.

**Figure 3:**
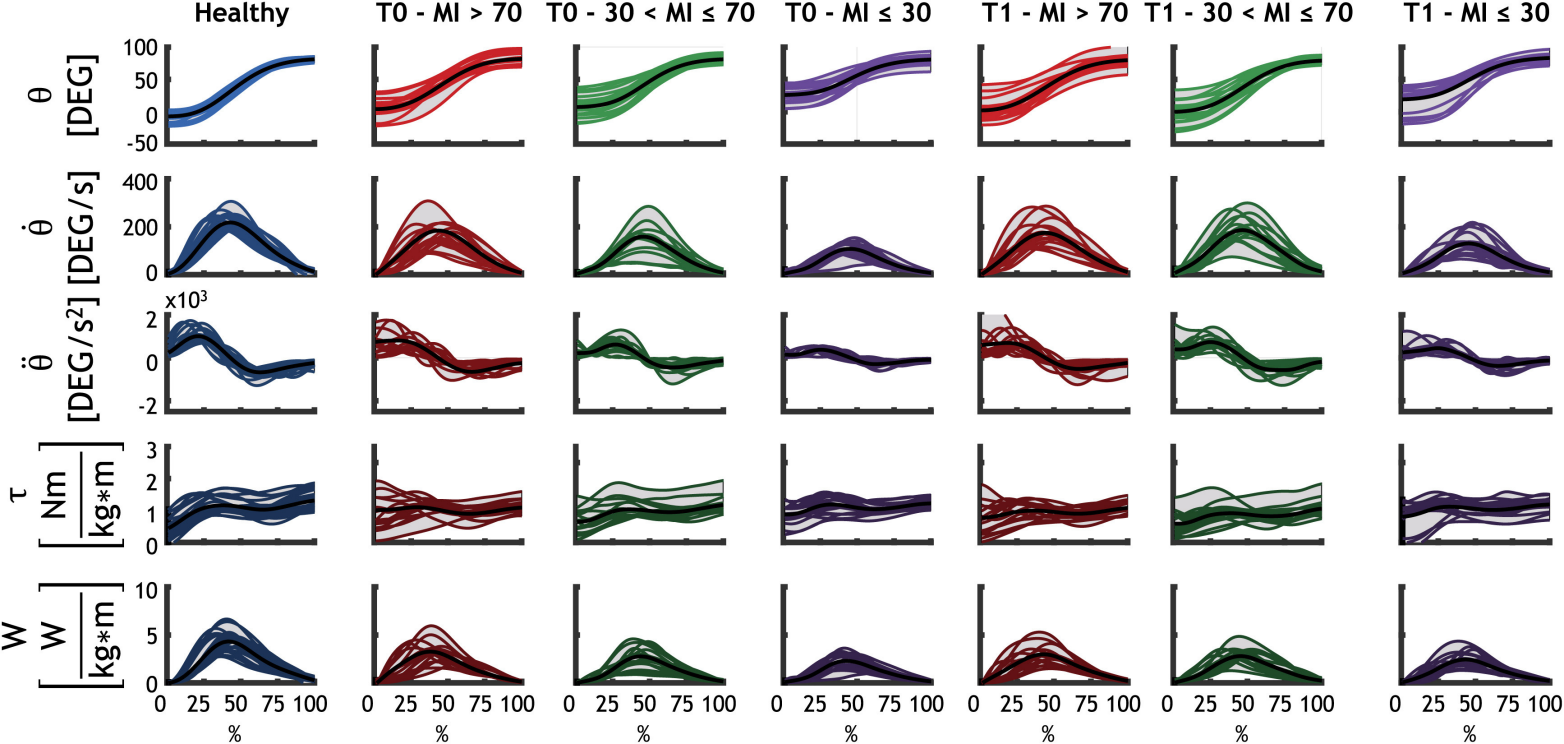
Detailed representation of the kinematics and dynamics of each group for the shoulder flexion. Each subject is represented with a line (as average for all the repetitions performed by that subject). The black line is the average of all subjects. Healthy subjects are represented with blue, mild patients (MI > 70) at T0 and at T1 are represented with red, moderate patients (30 < MI ≤ 70) at T0 and at T1 are shown in green, and severe patients (MI ≤ 30) at T0 and at T1 are shown in purple.

Figure 3 illustrates the kinematics, the dynamics and motor control indexes of the subjects' upper limbs. In the first three rows, the kinematics of each group is shown for the shoulder joint: angular position, velocity, and acceleration are portrayed. Dynamic parameters (including normalized torque and normalized power output) are shown in the last two rows.

**Figure 4:**
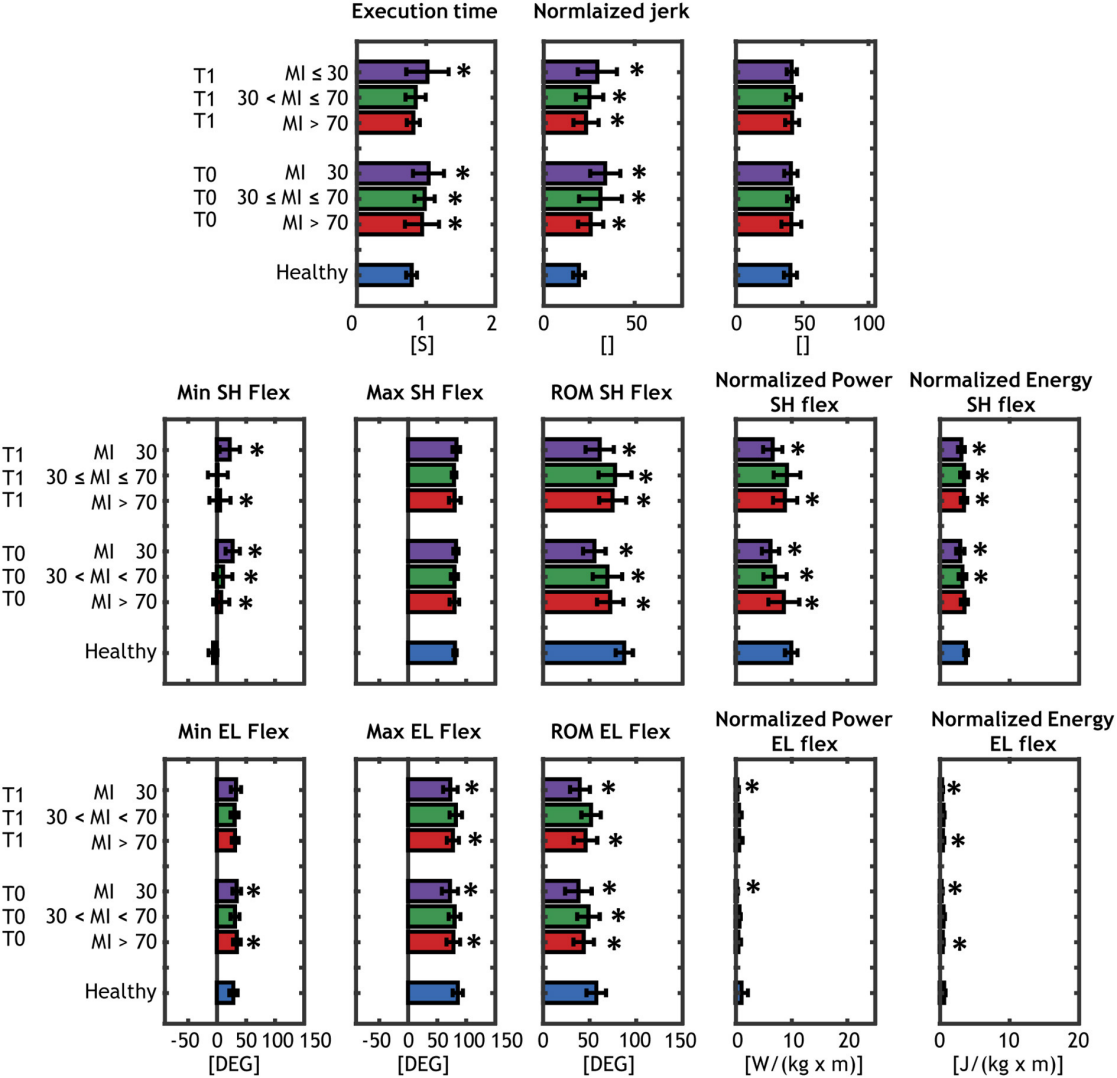
Results of the biomechanical assessment. Comparison between each of the patients' group (severe, moderate mild) at T0 and T1 and the age-matched controls are shown and matched with an asterisk when significantly different. Healthy subjects are represented with blue barplots, mild patients (MI > 70) at T0 and at T1 are represented with red barplots, moderate patients (30 < MI ≤ 70) at T0 and at T1 are shown in green, and severe patients (MI ≤ 30) at T0 and at T1 are shown with purple barplots.

The biomechanical performances of the groups of patients and controls are presented in Figure 4 and the results of patients are compared with the healthy controls in details in the following sections, based on the group subdivisions.

### Mild patients (MI > 70)

With respect to healthy individuals (mean = 0.79 s), the *TE* parameter was larger for mild patients in acute phase *TE* = 0.95 s (*p* = 0.008) and improved in the subacute phase *TE* = 0.82 s (*p* = 0.42). For motor control parameters, *NJ* was higher than controls (*p* < 10^−3^), while no significant differences were found in the *TP* parameter in both acute and subacute stage.

Mild patients showed a significantly higher *MIN*_*SF*_ in acute (*p* < 10^−5^) and subacute (*p* = 0.008) phase, while *MAX*_*SF*_ was very similar to the healthy controls. The total *ROM*_*SF*_ was smaller with respect to control group (*ROM*_*SF*_ = 87.6°) in both stages (acute, *p* = 0.0003; subacute, *p* = 0.003). *P*_*SF*_ and *E*_*SF*_ of mild patients were significantly lower than control subjects in both stages, except for *E*_*SF*_in acute phase (*p* = 0.08).

For elbow joint, *MIN*_*EF*_ and *MAX*_*EF*_ were very similar to controls and significant difference was found in acute phase for *MIN*_*EF*_ (*p* = 0.018) and in both stages for *MAX*_*EF*_(acute, *p* = 0.03; subacute, *p* = 0.016). The *ROM*_*EF*_ was lower in acute (*p* ≤ 0.001) and subacute (*p* = 0.006) phases. Both *P*_*EF*_ and *E*_*EF*_ were smaller than healthy group, but only *E*_*EF*_ showed significant differences (acute, *p* = 0.002; subacute *p* = 0.009).

### Moderate patients (30 < MI ≤ 70)

Moderate patients showed a significantly higher *TE* in the acute phase (*TE* = 0.95 s, *p* = 0.008) that improved in the subacute one (*TE* = 0.85, *p* = 0.12). As for mild group, moderate patients presented a higher *NJ* than controls (*p* < 10^−3^) and no significant differences in the *TP* parameter.

*MIN*_*SF*_ was significantly higher only in acute phase (*p* < 10^−5^), while *MAX*_*SF*_ was similar to the healthy group. *ROM*_*SF*_ was significantly reduced in both stages (acute, *p* < 0.001; subacute, *p* = 0.028). *P*_*SF*_ and *E*_*SF*_ of mild patients were significantly lower than control subjects in all stages.

Both *MIN*_*EF*_ and *MAX*_*EF*_were similar to healthy group and did not showed significant differences, while *ROM*_*EF*_ was significantly lower only in the acute phase (*p* = 0.043). No differences were found in dynamic parameters of the elbow joint (*P*_*EF*_ and *E*_*EF*_).

### Severe patients (MI ≤ 30)

Severe patients showed the highest differences in almost all the parameters. *TE* was 1.04 s (*p* < 10^−4^) in acute phase and did not improved in subacute phase (*TE* = 1.02 s, *p* = 0.001). *NJ* was significantly higher than controls (*p* < 10^−3^) and no significant differences in the *TP* parameter, as for the mild and moderate group.

*MIN*_*SF*_ was significantly higher in acute (*p* < 10^−5^) and subacute (*p* < 10^−5^) phase, while *MAX*_*SF*_ was similar to the healthy group. Severe patients presented the most reduced *ROM*_*SF*_ in both stages group [*ROM*_*SF*_ = 55.4° in the acute phase (*p* < 10^−5^) and *ROM*_*SF*_ = 61.1° in subacute phase (*p* < 10^−5^)]. *P*_*SF*_ and *E*_*SF*_ of severe patients were lower than control subjects in all stages.

For elbow joint, *MIN*_*EF*_ was significantly different only in the acute phase (*p* = 0.03), while *MAX*_*EF*_and *ROM*_*EF*_ were significantly lower in both phases (*MAX*_*EF*_ *p* ≤ 0.001; *ROM*_*EF*_ *p* < 10^−4^). Both *P*_*EF*_ and *E*_*EF*_ achieved by severe patients were lower than healthy control in acute (*p* = 0.01; *p* < 10^−5^) and subacute (*p* = 0.014; *p* < 10^−5^) phase.

## Discussion

In this study, we analyzed the performance of the ipsilesional limb in patients with stroke during multi-joint reaching tasks, comparing kinematic and dynamic data with controls. We showed that motor deficits in the ipsilesional side were present in all the groups of patients.

First, patients showed a tendency in performing slower movements. This result is in partial accordance with previous studies (32, 33), in which patients took longer time to complete reaching tasks in the horizontal plane. The smoothness of the trajectory (normalized jerk) was lower in all patients, indicating a difficulty in controlling the ipsilesional upper limb in multi-joint movements, probably due to altered activation and coordination of muscles (10).

Patients' elbow and shoulder joints showed limited ROM. We noticed that in severe patients, limited shoulder ROM was related not only to the lower maximum angle reached, but also to the higher starting angle, indicating that patients had a different postural accommodation that affected the range of motion. Dynamic measures provided further evidences of the motor control deficits of the ipsilesional upper limb during complex multi-joint movements in post-stroke patients. The energy and the power outputs were lower both in shoulder and in elbow joint with a high significance level.

Moreover, our study highlighted that higher ipsilesional motor control impairments were present in severe patients. This result agrees with previous studies (11, 15), that showed that motor impairment in the ipsilesional limb varied with the severity of the contralesional limb. Furthermore, patients reported higher motor deficits in the acute stage, regardless of the severity level. This finding proved that the motor impairment was more pronounced immediately after the stroke event (11).

Summarizing, significant difference was found between healthy people and patients' ipsilesional limbs when quantifying motion biomechanics. However, some points are still opened for further studies. First, we noted how some of the assessed parameters are partially dependent from the starting position. With the data in our possession, we cannot quantify whether these biomechanical differences reflect mainly a postural trunk control strategy or are intimately related to efficiency of the upper limb movement itself. However, since subjects were instructed to adhere at best to the proposed protocol (including the starting configuration), we conclude that trunk posture cannot be separated from the upper limb gesture and is by all means part of the assessment: in fact, it was demonstrated that trunk control posture is altered in stroke patients affecting motor performance (34). Furthermore, the effect of motivation was not tested in this study. It is indeed not quantifiable whether the effect of low-motivation can influence biomechanical performances (35). Recent studies demonstrated that better motor performances can be achieved if a subject is acting with the purpose of achieving specific aims (36). Moreover, improved motor performances were achieved in patients that were involved in a virtual reality environment (37) or receiving feedbacks from robot-assisted technologies (38). Under the effect of such drivers, we cannot exclude that motor performance might increase, comparing to our protocol including reaching movements at natural speed. It was also demonstrated with kinematic assessment that performing a well-learned upper limb movement with concurrent cognitive task leads to decreased efficiency of motor control in chronic stroke survivors (39).

Lastly, in this study, we did not investigate if the ipsilesional deficits were related to the damaged hemisphere. Previous studies (32, 33) showed that patients with the dominant hemisphere damaged had deficits in controlling the arm trajectory while non-dominant hemisphere damaged patients had problems in achieving the accurate final position. Their results supported the idea of the lateralization of motor control between the two hemispheres (40, 41) and that interhemispheric relationship may optimize motor control.

One limitation of our study was to verify the sensibility of a simplified protocol applied in clinical practice that employs a limited set of markers for tracking the kinematic data. However, this protocol already proved to be effective on healthy people (24) and on patients (17); in fact, we are able to detect relevant dynamic ipsilesional upper limb dysfunctions. The simplified protocol also matches well with the wide spreading use of low-cost sensors-based applications for motor evaluation in domiciliary context (18). Further studies could expand on this protocol by using more extensive kinematic models to increase its sensibility. Furthermore, we analyzed only the kinematics of each group, that in any case is related to intention of movement. Authors foresee the inclusion of electromyography to provide better insight into neuromuscular patterns at the motor control level.

## Conclusions

In this paper, we provide evidence that the ipsilesional upper limb of post-stroke patients shows motor deficits on kinematic, dynamic, motor control and energy consumption parameters during multi-joints movements. Patients present ipsilesional upper limb lower performances in the acute and subacute stage of the recovery. We found that patients with severe contralesional upper limb deficit are more impaired in terms of ipsilesional performances, suggesting to consider the ipsilesional limb in post-stroke patients to define their functional profile, to plan treatments and evaluate their efficacy. These results raise a need to further analyze the factors influencing the low performances of the ipsilesional limb in post-stroke patients.

## Data availability statement

The original contributions presented in the study are included in the article/supplementary material, further inquiries can be directed to the corresponding author/s.

## Ethics statement

The studies involving human participants were reviewed and approved by local ethical committee at ATS Insubria. The patients/participants provided their written informed consent to participate in this study.

## Author contributions

AS, EG, and FM: substantial contributions to the conception of the manuscript and methodology. AS, EG, RM, and CB: data curation and visualization. AS, RM, and CB: formal analysis and software. FM and AS: resources. AS, EG, LM, and FM: project administration. AS, LM, and FM: funding acquisition. All authors have participated to writing, reviewing, and editing the manuscript. All authors read and approved the final version of the manuscript.

## Conflict of interest

The authors declare that the research was conducted in the absence of any commercial or financial relationships that could be construed as a potential conflict of interest.

## Publisher's note

All claims expressed in this article are solely those of the authors and do not necessarily represent those of their affiliated organizations, or those of the publisher, the editors and the reviewers. Any product that may be evaluated in this article, or claim that may be made by its manufacturer, is not guaranteed or endorsed by the publisher.
